# Procoagulant and anticoagulant plasma indicators in diabetic dogs showing increased antithrombin III levels in canine diabetes mellitus

**DOI:** 10.1186/s12917-022-03179-7

**Published:** 2022-03-19

**Authors:** Andrzej Milczak, Dagmara Winiarczyk, Stanisław Winiarczyk, Diana Bochyńska, Łukasz Adaszek, Mateusz Winiarczyk, Roman Lechowski

**Affiliations:** 1 Department and Clinic of Animal Internal Diseases, Sub-Department of Internal Diseases of Small Animals, University of Life Sciences in Lublin, Lublin, Poland; 2 Department of Epizootiology and Clinic of Infectious Diseases, University of Life Sciences in Lublin, Lublin, Poland; 3 University of Cambridge, Queen’s Veterinary School, Cambridge, England; 4 Department of Vitreoretinal Surgery, Medical University of Lublin, Lublin, Poland; 5 Faculty of Veterinary Medicine, Department of Small Animal Diseases with Clinic, Warsaw University of Life Sciences, Warsaw, Poland

**Keywords:** Antithrombin III, Canine diabetes, Diabetes, Thrombosis, Blood coagulation

## Abstract

**Background:**

Diabetes mellitus (DM) often leads to dangerous thromboembolic complications in humans. DM is also a relatively common endocrinopathy of dogs. There is scarce information regarding procoagulant and anticoagulant plasma indicators in this disease. The aim of the study was to evaluate the levels of the selected plasma haemostatic parameters in dogs suffering from diabetes. The study group consisted of 20 dogs meeting all the inclusion criteria, with fasting glycaemia exceeding 11.1 mmol/l. The control group consisted of 15 healthy dogs presented for routine examination. An evaluation of the prothrombin time (PT); and fibrinogen, D-dimer and antithrombin III (ATIII) levels was performed.

**Results:**

Except for ATIII activity, the haemostatic parameter differences were not statistically significant. High values of ATIII activity were observed in 90% of diabetic dogs. On average, the values amounted to 166.6% and were 31.4% higher than those in the control group. The ATIII activity in the diabetic group was significantly higher than that in the control group (*p* = 0.0004).

**Conclusions:**

Here, we report elevated levels of ATIII in diabetic dogs. This finding may suggest the protective role of ATIII against potential thrombotic events. However, the exact role of ATIII in dog diabetes remains unclear.

## Background

Diabetes mellitus (DM) is a heterogeneous group of metabolic diseases characterised by the occurrence of chronic hyperglycaemia resulting from impaired insulin secretion or activity. The disease affects people and many different species of domestic and wild animals [1]. The estimated prevalence of diabetes in the canine population varies from 0.0013 to 1.2% [2–4]. In terms of the aetiology, pathogenesis, and course of the disease, canine diabetes is remarkably similar to type 1 diabetes in humans, insulin-dependent diabetes mellitus (IDDM) [2]. With insulin therapy, diabetes can be treated effectively, and with the proper control of glycaemia, long-term survival can be achieved. The mean survival time for dogs treated with insulin ranges from several months to 3 years [3, 5]. In humans, the survival rate is obviously much higher, but the risk of death increases dramatically with the onset of micro- and macrovascular complications in advanced DM [6–8]. Increased incidence of macrovascular complications such as ischaemic heart disease, deep vein thrombosis (DVT), and pulmonary embolism (PE) is commonly observed in human patients with diabetes [9–11]. Chronic hyperglycaemia plays a significant role in the pathogenesis of the abovementioned disorders. In humans, it has been demonstrated, that hyperglycaemia may be an important prothrombotic factor, although the manner in which hyperglycaemia leads to an increase in prothrombotic activity is not fully understood [10–14]. In diabetic human patients, increased concentrations of fibrinogen, factor VII, and fibrinolysis inhibitor (PAI-1) and decreased concentrations of natural anticoagulants (proteins C and S) are observed [13–15]. There is no consensus among researchers regarding the role of another important anticoagulant, antithrombin III (ATIII), during diabetes. Various studies conducted in human diabetic patients showed low, normal, or elevated plasma levels of ATIII [10, 13–19]. The authors of these publications did not find an explanation for the phenomenon of the increase in ATIII concentration over the course of DM. It is sometimes suggested that ATIII activity in the blood of diabetic patients increases, partially as a result of a compensatory mechanism [18]. Both the C, S and ATIII proteins regulate the plasma coagulation process, limiting the excessive formation of fibrin deposits at the site of damage to the vascular endothelium.

In the veterinary literature, there are few data on the incidence of thromboembolic events in dogs. We found only two studies documenting the occurrence of macrovascular complications of diabetes in dogs [20, 21]. A few older studies confirmed the presence of hypercoagulability syndrome in dogs, characterised by an increase in fibrinogen concentration; higher factor VII, VIII, X, XI, XII, and vWF activity; and a decrease in the concentration of the proteins C, S and ATIII [22–25].

The aim of the study was to evaluate the levels of selected plasma haemostatic system parameters and ATIII activity in dogs with newly diagnosed, untreated and uncomplicated DM.

## Results

Forty-two dogs of different breeds met the inclusion criteria for the study. Animals with Cushing’s syndrome (*n* = 5), pyometra (*n* = 6) and phaeochromocytoma (*n* = 1) were excluded from the study. Type I diabetes mellitus was diagnosed in the remaining 30 dogs. Of these patients, five animals with the coexistence of neoplastic disease and another five with abnormal CBC (complete blood count) or blood biochemistry values were excluded (*n* = 10). Ultimately, the study group included 20 dogs with uncomplicated and previously untreated diabetes. Breeds that were represented in this group included the Labrador (*n* = 2), Doberman Pincher (*n* = 1), German Shepherd Dog (*n* = 3), Cocker Spaniel (*n* = 5), and Polish Lowland Sheepdog (*n* = 2). Seven other dogs were mongrels. The proportions of male and female dogs were 46,7% (*n* = 7) and 53,3% (*n* = 8), respectively. The average age of diabetic animals was 11,26 (+/- 2,75) years.

Of the 15 healthy dogs from the control group, 60% (*n* = 12) were male and 40% (*n* = 8) were female. Eight of these animals were of various standard breeds (German Shepherd Dog (*n* = 4), Labrador (*n* = 1), Cocker Spaniel (*n* = 1), Fox Terrier (*n* = 1), Cocker Spaniel (*n* = 1)), and 7 were mongrel dogs. The average age of dogs from this group was 10,07 (σx 3,05) years. There were no significant differences between the control group and DM group in respect to age, sex, body weight and BCS.

The baseline laboratory characteristics of both groups are shown in Table 1. The results of Shapiro-Wilk test showed that most variables, except total protein in diabetic dogs follow normal distribution. The finding of the independent sample t-tests for WBC, RBC, PCV, HGB, and PLT showed a statistically significant difference among healthy and diabetic dogs (*p* < 0.05). There were no significant differences between the control group and DM group with respect to biochemical values, with the exception of total protein and fasting glucose level; however, for obvious reasons, this last parameter was not analysed.

**Table 1:** Basic laboratory data of tested dogs

Variables	Reference Interval	Data format	Control *n* = 15	Diabetic *n* = 20	Statistical significance of difference *p*
*CBC*					
WBC [× 10^9^/L]	5.0–14.1	Mean (SD)	7,49 (1,33)	10,42 (3,10)	**0,012**
RBC [× 10^12^/L]	4.95–7.87	Mean (SD)	7,46 (0,63)	6,61 (0,67)	**0,042**
PCV [%]	35–57	Mean (SD)	46,65 (3,91)	41,27 (5,61)	**0,011**
HGB [g/dl]	11.9–18.9	Mean (SD)	13,75 (1,54)	16,09 (1,73)	**0,002**
PLT [× 10^9^/L]	211–621	Mean (SD)	355,92 (72,82)	445,54 (140,08)	**0,034**
*Biochemical parameters*					
Fasting blood glucose [mmol/L]	3.3–6.2	Mean (SD)	< 11,11	18,54 (3,64)	n/a
Serum Urea [mmol/L]	3.5–9.0	Mean (SD)	5,99 (1,31)	7,44 (2,97)	0,099
Serum Creatinine [μmol/L]	20–150	Mean (SD)	63,65 (13,24)	54 (14,35)	0,071
Total Protein [g/dL]	55–74	Median (IQR)*	6,1(0,9)	6,8(0,30)	0,022**

Explanation: *p* < 0,05 significant difference; *n/a* not analysed

*Abbreviations*: *WBC* White blood cells, *RBC* Red blood cells, *PCV* Packed-cell volume, *HGB* Haemoglobin, *PLT* Platelets, *IQR* Interquartile range

^*^not normal distribution of variable

^**^analysed using Mann–Whitney U test

The selected parameters of the plasma haemostatic system are summarized in Table 2. Only PT and ATIII values in diabetic dogs were normally distributed. PT, fibrinogen and ATIII activity, were statistically different between groups, whereby PT in diabetic patients was lower than healthy dogs and fibrinogen, ATIII were higher compare to control group.. The lowest value of prothrombin time in DM group was 6.1 s. Fibrinogen levels were increased in 10 diabetic dogs and ranged from 326 to 1852 mg/dL. In the rest of the group, fibrinogen levels were within the physiological ranges. In terms of the concentration of D-dimers, the study group did not differ from the control group, but in 8 diabetic animals, the D-dimer concentration exceeded the cut-off point for normal values (500 μg/L), ranging from 512 to 1305 μg/L. High ATIII activity values were observed in 18 diabetic dogs (90%), exceeding the upper limit of the value range obtained in the control group by 31.6%.

**Table 2:** Values of the haemostatic parameters in control and diabetic dogs

Variables	Reference Interval	Data format	Control *n* = 15	Diabetic *n* = 20	Statistical significance of difference *p*
PT [s]	9–16	Mean (SD)	9,70 (1,49)	7,95(0,96)	**0,0034**
Fibrinogen [mg/dL]	100–250	Median (IQR)*	220 (84)	333(109,5)	0,0029**
D-D [μg/L]	≥250	Median (IQR)*	99 (119,5)	215(370,5)	0,0973**
ATIII [%]	65–145	Mean (SD)	135 (15,69)	166,6 (23,32)	**0,0004**

Explanation: *p* < 0,05 significant difference, *n/a* not analysed

*Abbreviations*: *ATIII* Antithrombin III, *PT* Prothrombin time, *D_D* D-dimer, *IQR* Interquartile range

^*^not normal distribution of variable

^**^analysed using Mann–Whitney U test

Age and glycemic level dependency for all tested coagulation parameters in DM group shows Table 3.

**Table 3:** Haemostatic indices and their correlation with age and fasting blood glucose level among diabetic dogs

Corelation	Spearman’s *ρ*
Age/PT	−0,477*
Age/Fibrinogen	0,335
Age/D-D	0,206
Age/ATIII	0,129
Blood glucose/PT	0,292
Blood glucose /Fibrinogen	−0,547
Blood glucose /D-D	0,138
Blood glucose /ATIII	−0,109

*Abbreviations*: *ATIII* Antithrombin III, *PT* Prothrombin time, *D_D* D-dimer

Explanation: * *p* = 0,015

There were also no statistically significant correlations between any of the analysed parameters.

## Discussion

The objectives of this study were to find evidence of the presence of haemostatic disorders in the course of canine DM. The obtained results partially fit into the spectrum of laboratory changes described in people suffering from DM. Moderate prothrombin time reduction, high levels of fibrinogen and high D-dimer values were observed in some patients. Similar changes occur in the different hypercoagulable states in humans [16, 26, 27]. Also, there were statistically significant differences in the blood morphology of our patients, commonly seen in the natural course of the diabetes. Currently, in human medicine, the terms thrombophilia and hypercoagulability are often used interchangeably to describe a genetically determined or acquired susceptibility to venous or arterial thrombosis associated with haemostatic abnormalities [28]. In the broadest sense, a hypercoagulable state is any acquired or inherited condition that increases the risk of thromboembolism. In diabetic persons, especially those suffering from type 2 diabetes, imbalances in plasma procoagulant/anticoagulant activity can be observed. DM causes atherosclerosis, and approximately 80% of patients die from thrombotic complications [14, 29]. Studies conducted in relatively small groups of children with type 1 diabetes demonstrated the occurrence of laboratory features of hypercoagulation but no clinical changes suggestive of thrombosis [26]. We found only two reports regarding the occurrence of arterial and venous thrombosis in diabetic dogs. The authors of the first publication described 31 cases of dogs with abdominal aorta thrombosis. The primary cause of thrombosis was mostly kidney disease. Diabetes was indicated as the cause of thrombosis in just one case [20]. The authors of the second publication described 80 dogs with splenic vein thrombosis, with only one case of diabetes concomitant with pancreatitis [21].

According to the authors, the use of the term “thrombophilia” when referring to dogs suffering from diabetes is not justified at the current state of knowledge. Neither the literature review nor the results of our research provide evidence of an increased incidence of thrombosis in diabetic dogs. Although increased fibrinogen concentration and shortened PT in dogs may be indicative of a hypercoagulable state [30], the presence of increased circulating D-dimer concentration was not statistically confirmed in the DM group. Incidental changes in this parameter can be explained in a variety of ways. Increased concentrations of D-dimers can be, for instance, observed during inflammation, which is often present in diabetic patients [9, 31]. Hyperglycaemia is a pro-inflammatory factor. An increased blood glucose level leads to impaired endothelial nitric oxide synthase (eNOS) activity and increased production of reactive oxygen species (ROS), as well as the release of pro-inflammatory cytokines, such as Il-1 and Il-6 TNF-α 8. Statistically higher concentrations of fibrinogen in dogs from the DM group seem to confirmthese findings, as inflammation is the most common cause of hyperfibrinogenemia.

In human medicine based on the standard coagulation parameters a numerous clinical thromboembolic prediction scores have been developed, however their reliability remains uncertain [32]. Recently, high hopes are placed on a global whole-blood viscoelastic hemostatic tests. It is considered that an increased in vitro clot strength, demonstrated on a viscoelastic test, reflects a hypercoagulable state [26, 32]. Indeed, a hypercoagulable pattern of haemostasis was reported in many patients suffering from diseases with a documented risk of thrombosis. Similar changes were also identified in children with type 1 DM [26]. We also obtained similar results in some diabetic dogs using ROTEM assay (rotational thromboelastometry). A preliminary study of 6 diabetic dogs reveled that, in the extrinsically activated thromboelastography (ExTEM) assay the maximum clot firmness (MCF) was significantly (*p* = 0,0024) higher (78,67 ± 2,52 mm) than in the control group (65,0 ± 3,0 mm) (unpublished data). Nevertheless, there are some limitations to viscoelastic assays. First, the authors of a recent meta-analysis concerning the ability of viscoelastic tests to identify a hypercoagulable state in peoples confirmed the low sensitivity of such assays. Not all patients who developed thromboembolic events could be identified by this tests [32]. Second, global haemostasis tests do not allow to fully elucidate the etiopathology of coagulation disorders, as they do not identify individual components of the haemostatic system. Surprisingly, in our study, we found elevated levels of ATIII in diabetic dogs. Only 2 dogs with DM showed slightly lower (by 10%) ATIII values than the highest ones observed in the control group. In the entire study group, the average value of ATIII activity was 31.4% higher than that in the control group.

A similar phenomenon occurs in humans, and to some extent, in dogs with Cushing’s syndrome, which is known as the prothrombotic state [33]. It has been suggested that a high risk of thrombosis is observed with ATIII activity values below 60% [34]. This risk decreases with normal ATIII values. One would expect that a high activity of ATIII should be associated with a reduced thrombotic risk. The absence of obvious laboratory features of hypercoagulability together with high ATIII activity found in our study support this theory.

Antithrombin III is a single-chain glycoprotein belonging to the family of serine proteases, so-called serpins. Antithrombin III is synthesised mainly in the liver but also in vascular endothelial cells, megakaryocytes, and platelets. The half-life of ATIII is quite short: in dogs, it is 1.7 days [22]. The main role of ATIII is to inhibit the coagulation system. Antithrombin III inactivates thrombin as well as the following factors: Xa, XIIa, XIa, IXa, and, in the presence of heparin, factor VIIa [15, 17]. In addition to inhibiting the coagulation system, ATIII also exerts anti-inflammatory effects. ATIII is also considered a negative acute phase protein [35].

The majority of human studies show normal [14] or reduced [12, 15, 17, 29] ATIII levels in diabetic people. Most of the studies were conducted in patients with type 2 diabetes. However, the authors of several reports found high ATIII levels in patients with type 1 diabetes [13, 18, 19]. As already mentioned, canine diabetes is similar to type 1 diabetes in humans.

Certain analytical methods are prone to interference from other analytes. The presence of some protease inhibitors in the sample may be the cause of a false increase in ATIII activity. Such proteins include heparin II (HCII) cofactors and alpha2-macroglobulin. The latter may be responsible for 10–25% of the antithrombin plasma activity. The ATIII determination method using the chromogenic substrate and factor Xa is not sensitive to this type of interference [36]. By using this method in our research, we avoided the potential analytical error.

Genetic factors most likely play a major role in the pathogenesis of macrovascular complications in type 1 diabetes. For example, it has been shown that the incidence of acute episodes of ischaemic heart disease increases in patients with some haptoglobin allotypes or HLA antigens [37]. It cannot be ruled out that the varied results of ATIII activity obtained by researchers from different countries result from genetic differences of populations studied by them. A similar hypothesis was made by Hamulu et al. They pointed to high ATIII levels in human diabetic patients treated in Izmir [18]. An interesting fact, which seems to confirm such a possibility in relation to various dog populations, was found in one of the publications on diabetes in dogs [3]. The authors pointed out a significant difference in the survival rate of dogs suffering from diabetes in Great Britain and Sweden. Even though both countries have similar socio-economic levels, the mean survival time from the day of diagnosis for dogs in England was 17.3 months, while in Sweden, it was only 57 days. Of course, on the basis of this information alone, it cannot be confirmed that such a significant difference results from the genetic differences of the studied populations, but such a possibility is probable.

Some authors have tentatively attributed this increased activity of natural anticoagulants to the compensatory response to the prothrombotic processes. Yet the fact remains that elevated activity of ATIII may be observed in diabetic dogs. This should be taken into account in the diagnostic and therapeutic strategy.

## Conclusion

To our knowledge, this is the first report describing the presence of elevated plasma levels of antithrombin III in diabetic dogs. One may hypothesise that high antithrombin activity plays a protective role against excessive coagulation activation [18]. To confirm this, larger studies taking potential populational differences into account should be conducted to provide fully reliable results.

## Material and methods

### Study design and setting

The study was performed in 42 dogs treated in two large veterinary private clinics from western Poland and the Innovative Center for Animal Pathology and Therapy of Faculty of Veterinary Medicine in Lublin. The estimated number of dogs treated in these 3 units was over 40,000 per year, and the percentage of newly diagnosed cases of diabetes did not exceed 0.25% of all patients.

#### Study group

The study included dogs admitted to the clinic with symptoms suggestive of DM. The main clinical signs observed in all dogs at admission were weakness, polyuria and polydipsia. The control group consisted of 15 healthy dogs presented for prophylactic examinations before vaccination. CBC and main blood biochemistry parameters of the control group were within the physiological range. Informed consent was obtained from the owners prior to clinical investigations and sample collection.

#### Inclusion and exclusion criteria

Dogs were eligible for the study if they met all the following inclusion criteria: presence of polyuria and polydipsia, fasting blood glucose level ≥ 11,1 mmol/l (200 mg/dl) at admission and glucosuria. Animals with previously diagnosed and already treated diabetes or those with hyperglycaemia caused by endocrinopathies other than hypoinsulinaemia (e.g., pyometra, Cushing’s syndrome) as well as dogs with concurrent neoplastic disease were excluded. The diagnosis of these conditions were made based on clinical, clinicopathological, specific endocrine tests and diagnostic imaging findings where appropriate.

In addition, as the exclusion criteria, the following states were considered: active infections with leukocytosis, moderate or severe anaemia, thrombocytopenia, chronic kidney disease, and liver injury, presenting with hypoalbuminemia, elevated serum alanine transaminase and bilirubin levels. Values of WBC > 20 × 10^9^/L, PCV < 30%, PLT < 150 × 10^9^/L, blood urea > 28,80 mmol/L, ALT > 200 U/L, Alb < 2,8 mg/dL, bilirubin > 0,6 mg/dL and urine protein to creatinine ratio (UP:UC) > 0,5 were also an exclusion criteria.

#### Blood sample collection

Blood from the cephalic vein was collected from each dog in the sitting position using vacuum system tubes containing K2EDTA (1 ml), clot activator (2 ml), NaF/EDTA (2 ml) and 3.2% buffered sodium citrate solution (1,8 ml). Before sampling, the skin was shaved, and the whole procedure was carried out under aseptic conditions.

##### Haematology, biochemistry, urinalysis and hormones measurements

Blood was collected in EDTA and non-EDTA tubes from each dog in the sitting position from the cephalic vein using a vacuum system. All the following procedures were carried on as in our previous experiment [38]. In detail, before sampling, the skin was shaved, and the whole procedure was carried out under sterile conditions. A complete blood count (CBC) was performed for each dog with an Exigo Vet analyser (Boule, Spånga, Sweden). The serum obtained after centrifugation at 1500×g for 10 min in a refrigerated centrifuge was analysed in a BS-130 automatic biochemical analyser (Mindray, Shenzhen, China). The chemistry panel included alanine transferase (ALT), aspartate aminotransferase (AST), total bilirubin, urea, creatinine, alkaline phosphatase (AP), albumin, and total protein (TP). The blood glucose concentration was measured using EDTA-anticoagulated whole blood with the addition of sodium fluoride (NaF). Voided midstream urine samples were collected in the morning and were subjected to complete routine urinalysis with sediment examination and quantitative assessment of proteinuria using the urine protein to creatinine ratio (UPC). Urine total proteins and creatinine were determined using commercial kits on an automated chemistry analyser (Mindray BS-130). The UPC was calculated using the following formula: UPC = urine protein (mg/dL)/urine creatinine (mg/dL). The whole sample preparation procedure was performed within 1 h of material collection. In justified cases, the concentration of the serum cortisol using enzyme-amplified chemiluminescent assay (Immulite 1000 analyzer) was determined.

### Coagulation methods

The selected parameters of the plasma haemostasis system, namely, prothrombin time (PT), concentration of fibrinogen, and D-dimers as well as antithrombin III (ATIII) activity, were determined using a Bio-Ksel 6000 automated coagulometric analyser (Bio-Ksel, Poland). Citrate-anticoagulated platelet-poor plasma obtained within 15 min from blood collection was used for the analysis. Pooled human plasma was used to calibrate the coagulometer. The fibrinogen concentration was determined using a PT-derived assay. The D-dimer concentration was determined quantitatively using immunoturbidimetry, and the ATIII activity was measured by means of the amidolytic method using a chromogenic substrate.

### Statistical analysis

Laboratory results of all parameters were pooled for statistical analysis. Data distribution was tested using the Shapiro-Wilk test. Parametric variables were expressed as mean ± standard deviation while non-parametric variables were expressed as median and interquartile range. For the measured parameters, independent sample t-tests and Mann-Whitney U tests were performed to compare the differences between the DM and control groups. Post-hoc analysis of the power of tests used in statistical analysis was evaluated at p level = 0,05. The Pearson correlation coefficient (Pearson r) was used to test the correlation between such variables as age of the animal or blood glucose and coagulation parameters (PT, fibrinogen, ATIII, D_D). Due to the relatively small number of observations, the relationships between parameters were also assessed by means of a two-tailed Spearman’s rank correlation test. A *p* value < 0,05 was considered statistically significant. All statistical analyses were performed using the computer software PQStat v.1.8.2. (PQStat Software, Poznań, Polska).

## Data Availability

The datasets used and/or analysed during the current study are available from the corresponding author on reasonable request.

## Abbreviations

DM: Diabetes mellitus
IDDM: Insulin-dependent diabetes mellitus
ATIII: Antithrombin III
PT: Prothrombin time
CBC: Complete blood count
D_D: D-dimers
DVT: Deep vein thrombosis
PE: Pulmonary embolism
TP: Total protein
ALT: Alanine transferase
AST: Aspartate aminotransferase
UPC: Urine protein to creatinine ratio
eNOS: Endothelial nitric oxide synthase
